# Facile and noninvasive passivation, doping and chemical tuning of macroscopic hybrid perovskite crystals

**DOI:** 10.1371/journal.pone.0230540

**Published:** 2020-03-17

**Authors:** Ahmad R. Kirmani, Ahmed E. Mansour, Chen Yang, Rahim Munir, Ahmed M. El-Zohry, Omar F. Mohammed, Aram Amassian

**Affiliations:** Physical Sciences and Engineering Division (PSE), King Abdullah University of Science and Technology (KAUST), KAUST Solar Center (KSC), Thuwal, Kingdom of Saudi Arabia; University of Salento, ITALY

## Abstract

Halide vacancies and associated metallic lead (Pb°) observed at the surface and deep inside macroscopic organolead trihalide perovskite crystals is removed through a facile and noninvasive treatment. Indeed, Br_2_ vapor is shown to passivate Br-vacancies and associated Pb° in the bulk of macroscopic crystals. Controlling the exposure time can markedly improve the overall stoichiometry for moderate exposures or introduce excessive bromide for long exposures, resulting in *p*-doping of the crystals. In the low dose passivation regime, Hall effect measurements reveal a *ca*. 3-fold increase in carrier mobility to *ca*. 15 cm^2^V^-1^s^-1^, while the *p*-doping increases the electrical conductivity *ca*. 10000-fold, including a 50-fold increase in carrier mobility to *ca*. 150 cm^2^V^-1^s^-1^. The ease of diffusion of Br_2_ vapor into macroscopic crystals is ascribed to the porosity allowed in rapidly grown crystals through aggregative processes of the colloidal sol during growth of films and macroscopic crystals. This process is believed to form significant growth defects, including open voids, which may be remnants of the escaping solvent at the solidification front. These results suggest that due to the sol-gel-like nature of the growth process, macroscopic perovskite crystals reported in this study are far from perfect and point to possible pathways to improving the optoelectronic properties of these materials. Nevertheless, the ability of the vapor-phase approach to access and tune the bulk chemistry and properties of nominally macroscopic perovskite crystals provides interesting new opportunities to precisely manipulate and functionalize the bulk properties of hybrid perovskite crystals in a noninvasive manner.

## 1. Introduction

Hybrid organic-inorganic perovskites have recently emerged as strong contenders for next generation thin film optoelectronics, owing to their interesting charge transport properties and their ease of solution-processability.[1–5] A spate of breakthroughs in device designs and thin film preparation protocols in the last few years has helped the perovskite solar cells community to achieve >23% power conversion efficiency (PCE).[4] Additional, concurrent advances have been made in the successful utilization of hybrid perovskites as light emitting diodes and photodetectors.[6–8]

It is, however, difficult to completely avoid residual chemical contamination and defects in perovskite thin films, given the realities of solution-processing and the sol-gel nature of hybrid perovskite solidification.[9, 10] The presence of defects and contamination on surfaces is very much expected, but these have been believed unlikely to be present within the bulk of macroscopic single crystals, as the latter are considered the most pristine embodiments of the material.[11] Macroscopic single crystals of methylammonium lead tribromide (MAPbBr_3_) have been shown to exhibit a low density of trap states,[12] while polycrystalline thin films of the same chemical compositions suffer from several extrinsic factors such as grain boundaries, which also play a role in defining charge transport. Macroscopic crystals therefore provide a model surface which resembles that of thin films,[13, 14] along with a far more pristine bulk than the latter. However, recent reports have questioned the pristine quality of macroscopic crystals, suggesting that the electronic properties of hybrid perovskites are only modest at best, compared to gallium arsenide and silicon.[15, 16] It has been suggested that the internal structure of the MAPbBr_3_ crystals is far from perfect due to light- and environment-induced macroscopic voids.[17]

Herein, we find that as-synthesized macroscopic MAPbBr_3_ crystals exhibit significant presence of chemical contaminants such as oxygen and amorphous carbon, along with a substantial amount of metallic lead (Pb°), both at their surface and—most intriguingly—deep in the bulk. The Br:Pb ratio is 2.6, instead of 3. We access the crystal’s bulk by cleaving and examining its fresh surface, and find that Pb° and contaminants are still present, albeit to a lesser but still notable extent. We devise a simple, single-step, post-synthesis Br-vapor treatment to undo some of these ill-effects at the surface, but to our surprise, we find that the vapor permeates through the bulk crystal, increasing the Br:Pb ratio and demonstrably suppressing Pb° both at the surface and deep inside the bulk. The bulk incorporation of bromine from the vapor phase, as confirmed by X-ray diffraction (XRD), X-ray fluorescence (XRF) and X-ray photoelectron spectroscopy (XPS), points to significant pathways for diffusion and permeation, which we ascribe to growth imperfections and voids associated to the colloidal sol-gel nature of self-assembly/crystallization in organolead perovskites. Porosity in these crystals is also a likely outcome of halide vacancies. Interestingly, the presence of oxygen vacancies is a well-known observation in oxide perovskites, and is being investigated for potential gas (for e.g. carbon dioxide) adsorption applications.[18–20] Access to the bulk of macroscopic crystals by a Br_2_ vapor meant that the Br content could be tuned post-growth. Pb° was shown to be passivated after relatively short vapor exposures by careful XPS measurements, while longer exposures led to *p*-doping of the crystal with bromide. In the latter case, a remarkable 10000-fold enhancement in bulk electrical conductivity was measured, including a 50-fold increase of mobility up to ~150 cm^2^/Vs. The significant improvement in bulk transport properties provided further proof and points to the presence of Pb° as an important obstacle to charge transport in hybrid perovskite semiconductors. The opportunity to non-invasively access the bulk properties of macroscopic semiconductor crystals, previously thought to be compact and impermeable, opens up new opportunities for tuning the semiconductor and optoelectronic properties as well as adding new functionalities to this important class of semiconductors after growth.

## 2. Materials and methods

### Perovskite single crystal synthesis

The single crystals were synthesized using the inverse temperature crystallization protocol reported elsewhere.[12, 21] Lead bromide (99.99%) and dimethylformamide, DMF (anhydrous, 99.8%) were purchased from Sigma Aldrich. Methylammonium bromide (MABr) was purchased from Greatcell Solar (Australia). All salts and solvents were used as received without any further purification. 1.5 M PbBr_2_ and MABr was prepared in DMF. The solutions were filtered using a PTFE filter with 0.2 mm pore size. Two mL of the filtrate were placed in a vial and the vial was kept in an oil bath, increasing the temperature from 60 °C to 100 °C. The crystals were grown for 4 h. We have chosen MAPbBr_3_ single crystals over the other types, since the stability of these crystals has been demonstrated in the ambient.[22] MAPbI_3_ crystals are known to degrade in ambient conditions.[23] The crystals were *ca*. 5 mm x 3 mm x 2 mm in dimensions. We note that the proposed porosities/voids that allow bromine vapors to enter the crystals, are an outcome of the aggregative assembly involved in perovskite crystallization, whereby the sol, consisting of bromoplumbate-solvent complexes aggregates and solidifies without full densification and introduces unintentional voids into the crystals.

### Single crystal cleaving

The perovskite single crystal was cleaved in ambient and N_2_ conditions using a steel blade that scraped off the top few millimeters of the crystal. Thickness of each crystal was measured through Vernier calipers. Thickness of 1.27 mm, 0.69 mm and 1.15 mm was recorded for the as-is, brominated and cleaved crystals, respectively.

### X-ray photoelectron spectroscopy

XPS was measured in an ultrahigh vacuum (UHV) Omicron chamber equipped with a SPHERA U7 hemispherical energy analyzer. Photoemission was carried out using X-ray photons with an incident kinetic energy of 1486.6 eV obtained from a monochromated Al Kα X-ray source with a total energy resolution of 0.1 eV. The chamber base pressure for these measurements was < 5 × 10^-9^ mbar. Spectra were collected at room temperature. Importantly, all the high-resolution core levels (including Pb 4f) were collected within 10 min of X-ray exposure to avoid measurement artefacts. The X-ray flux, exposure time, and the UHV conditions were kept exactly the same throughout the study, across all the samples. The X-ray spot size was in the order of microns. XPS measurements were calibrated using the Fermi edge of a sputter-cleaned Ag sample. Relative atomic quantification was carried out using the CasaXPS software by comparing the relative intensities of the various core level peaks, employing appropriate relative sensitivity factors (RSFs). RSFs take into account the different differential cross sections, electron transmission and asymmetry parameters for the different orbitals. Shirley background subtraction was used for peak fitting.

### Kelvin probe measurements

The contact potential difference (CPD) was measured by Kelvin Probe (KP technology Ltd., UK) with reference to a vibrating stainless-steel tip of 5 mm diameter. The reported values were averaged over 100 readings for each measurement. The measurements were carried out in a nitrogen glove box environment. An HOPG sample was used for calibration.

### Hall effect measurements

Room-temperature Hall effect measurements were performed using the van der Pauw method in a commercial Lakeshore 7700 system with linear sweeps of magnetic fields up to 2 T using a 20 nA excitation current. Contacts were fabricated using conductive silver paste to fix low strain Ag alloy wires (Lake Shore PN 671–260) which were soldered to the sample holder.

### XRD measurements

XRD measurements were carried out at a Bruker D8 Advance XRD equipment equipped with Cu K-alpha source. Reflected scattering from the crystals was measured. Height alignment was performed for all samples and so the observed peaks shifts are not due to subtle differences in the sample heights/thicknesses.

### Bromine-vapor treatment

MAPBBr_3_ crystals were suspended in a sealed container holding liquid bromine kept at room temperature (approximately 5 cm above liquid Br_2_). Br_2_-vapor treatment was carried out in an ambient of uncontrolled humidity (ca. 50–60% RH) at room temperature, ca. 22 °C. We did not perform a direct measurement of the vapor pressure of bromine, but we believe the vapor pressure saturates in the sealed container achieving its room temperature of 270 mbar.[24] The bromine uptake by the crystals was controlled simply by the exposure time inside the sealed container.

### Photoluminescence measurements

PL spectra were collected in air by exciting the crystals with a Cobalt solid-state laser operating at 473 nm, with excitation neutral density filter (ND 2.0). The emitted light was collected into a spectrometer equipped with 600/600 grating and recorded using a Peltier-cooled CCD camera (Andor) using a 1 second acquisition time.

### UV-vis absorption measurements

Absorbance was measured using Varian Cary 5000 UV-vis spectrometer, using a transmission mode in the range of 450–800 nm. All measurements were taken with respect to air as baseline.

### X-ray fluorescence measurements

XRF spectra were collected using Bruker M4 Tornado. The X-ray tube (Rh) was set at 50 kV and 280 μA, and the signal was collected using XFlash430 detector. The measurements were performed at low vacuum conditions (~2 mbar).

### Time Correlated Single Photon Counting (TCSPC)

The sample was excited by pulsed laser diode (400 nm) that was purchased from HORIBA Jobin Yvon, model (DD-405L, IRF ≈ 65 ps). The repetition rate of the pulsed laser ranges from few kHz to MHz by DDC1 picosecond controller purchased also from HORIBA. The laser light is tightly focused on the sample using objective lens brought from Olympus Ltd., (Plan N, 10x/0.25). The maximized emission is collected from the same excited area by the objective lens after passing through reflective beam splitter (R 488), bought from Thorlabs. Then, the collected emission was focused on a commercial Avalanche photodiode that is controlled and by Hydra Harp 400 multichannel picosecond event timer unit, purchased from PicoQuant, to detect the emission in a single photon fashion, i.e., one per laser pulse at max. Then, the collected emission intensity versus time was analyzed and fitted using multi-exponential decay equations.

## 3. Results and discussion

We employed X-ray photoelectron spectroscopy (XPS) to study the surface and bulk chemical compositions of macroscopic MAPbBr_3_ crystals, synthesized by the inverse temperature crystallization technique (S1 Fig).[12] To compare the surface and the bulk, we cleaved the macroscopic crystal in an inert environment and compared the properties of this newly formed pristine surface (called ‘*cleaved*’ hereafter) with those of the native surface of as-synthesized crystals (called ‘*as-is*’ hereafter). We note that although XPS is primarily a surface-sensitive technique with up to 10 nm depth sensitivity, measurement of the freshly *cleaved* surface is only an indirect way to examine the bulk. In other words, the near-surface region of a *cleaved* crystal is expected to resemble the bulk, ignoring, for the context of this study, the various surface relaxation and reorganization phenomena which are confined to the topmost monolayers, and which are outside the current scope. This approximation has recently been used by Snaith and Koch groups which have utilized XPS on cleaved MAPbBr_3_ and methylammonium triiodide (MAPbI_3_) crystals to probe dopant concentration and the impact of light illumination, respectively, in the crystal bulk.[25, 26] In principle, hard X-ray photoelectron spectroscopy (HAXPES) measurements can allow for a deeper sample probing due to larger inelastic mean free paths of the photoelectrons at higher kinetic energies, these need to be carried out at a synchrotron facility and, as such, are beyond the scope of this study.[13]

### 2.1. Presence of Pb°

**Fig 1:**
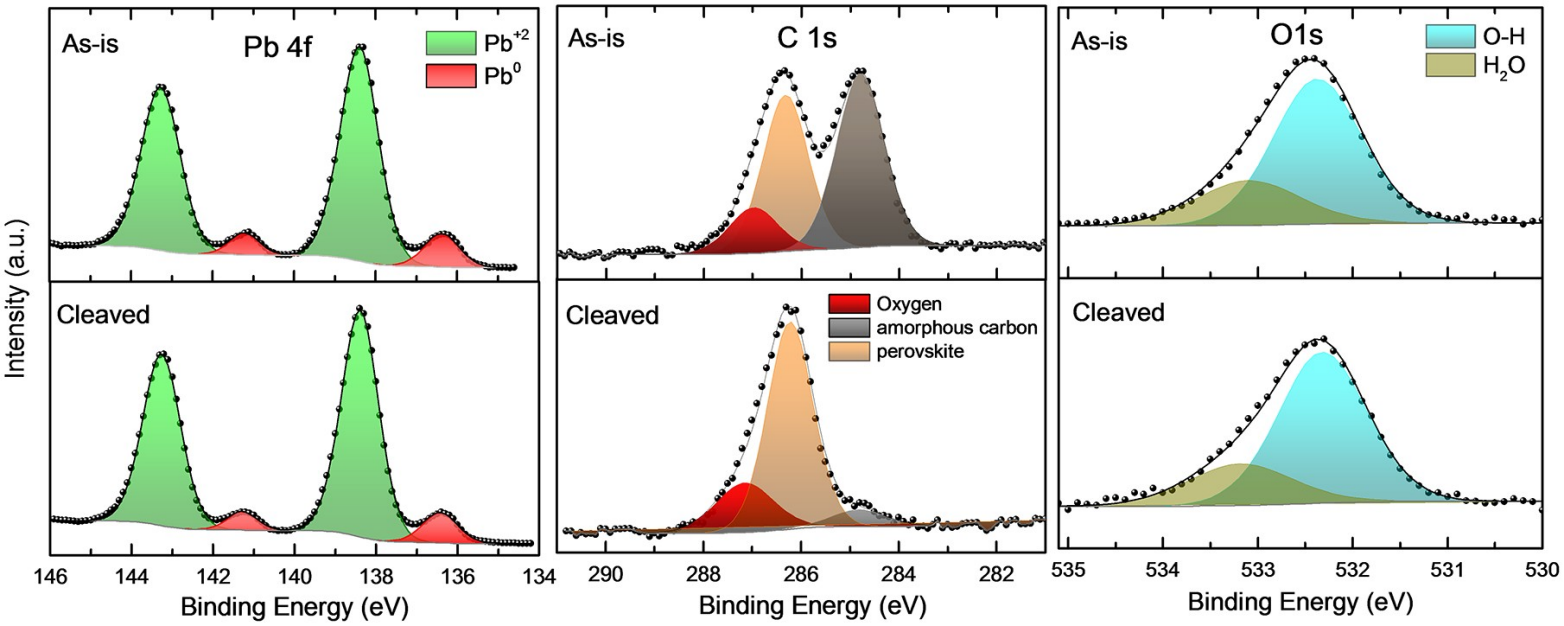
Presence of Pb° and chemical contaminants. XPS data showing the Pb 4f, C 1s and O 1s core level peaks for the *as-is* and *cleaved* surfaces of crystals. Pb 4f peaks show a shoulder (red) *ca*. 2 eV below the main peaks (green), corresponding to Pb°. C 1s core peaks can be split into three components: amorphous carbon (grey), carbon belonging to methylammonium (orange), carbon belonging to oxygen-functionalities (red). Oxygen functionalities, depicted by O 1s comprise of O-H (blue) and adsorbed water (green) components. Possibly, the O-C species overlaps the O-H component.

XPS spectra of the Pb 4f core level for the *as-is* and *cleaved* surfaces are shown in Fig 1. The Pb 4f core level peaks of the *as-is* surface reveals that in addition to the major components, namely the 4f_7/2_ and 4f_5/2_ doublet (138.4 and 143.3 eV, respectively), we observe shoulders at lower binding energy (BE), (136.4 and 141.3 eV, respectively). These have recently been ascribed to Pb° due to under-coordinated Pb.[7, 26–29] We find the Br:Pb ratio (*ca*. 2.6) to be below the stoichiometry of the perovskite at the *as-is* surface, pointing toward the presence of PbBr_2_. The C 1s core level peak on *as-is* surfaces, is found to comprise of 3 components: amorphous carbon, carbon belonging to CH_3_NH_3_ and a higher BE component (*ca*. 287 eV) corresponding to carbon-oxygen functionalities. We posit that the amorphous carbon is from residual solvents used during the crystal growth process. A significant amount of oxygen is also found on *as-is* surfaces.[30, 31]

It is important to note that MAPbBr_3_ crystals tend to be robust during brief XPS measurements and do not undergo any measurable chemical changes, as recent, carefully performed studies have shown.[26, 32] Whereas brief exposures to X-rays do not induce any noticeable changes,[26] longer exposures up to 1.5 hours cause MAPbBr_3_ to degrade causing a very small, but noticeable increase in Pb°.[32] Keeping the vulnerability of MAPbBr_3_ to long X-ray exposures in mind, we have performed XPS measurements on freshly prepared and cleaved crystals strictly keeping the X-ray exposure times to within 10 minutes for all the Pb 4f scans. It was also ensured that X-ray flux, exposure time and the ultra-high vacuum (UHV) conditions remained the same across all the samples measured. S2 Fig. shows a high resolution Pb 4f scan taken on the same spot on a cleaved crystal 1 hour apart during constant X-ray exposure under normal measurement conditions. No change in peak shape and position is detected confirming the robustness of our crystals to the X-ray exposure conditions. We are therefore able to confidently ascribe the observed Pb° as intrinsic to the crystals and not a measurement artifact.

The chemical impurities and metallic lead detected on *as-is* surfaces also abound in the bulk of the crystal, as revealed by XPS measurements on *cleaved* surfaces (Fig 1, lower panel and Table 1), whether prepared in air or in inert atmosphere (S1 Table). The Pb° content is found to remain high, but decreased slightly by *ca*. 12% compared to the *as-is* surface. C:Pb and O:Pb ratios are reduced from 2.9 and 0.7 for the *as-is* surface to 1.3 and 0.3 upon *cleaving*. The Br:Pb ratio remains relatively unchanged (*ca*. 2.5), suggesting that Br-deficiency prevails throughout the crystal. The *cleaved* surface, however, does show a significantly reduced amorphous carbon content, suggesting the residual solvent is primarily present near the growth surface of the crystal. Importantly, the ratio of the CH_3_NH_3_^+^ carbon component in C 1s (orange component of C 1s in Fig 1) to Pb is similar for both the *as-is* and *cleaved* surfaces (0.95), suggesting a uniform presence of the methylammonium cation across the surface and the bulk.

**Table 1:** Summary of atomic ratio determined from XPS relative atomic quantification.

Sample	Atomic ratio			
	Br:Pb	N:Pb	C:Pb	O:Pb
As-is	2.6	1.1	2.9	0.7
Cleaved	2.5	1.0	1.3	0.3

The likely presence of PbBr_2_ in crystals can be explained by chemical reactions between perovskite and the ambient moisture. Perovskites are prone to hydration,[33] as summarized for the well-known case of MAPbI_3_ in Eq (1):

$$4{\text{MAPbI}}_{3}\left(\text{s}\right)\;+\;2{\text{H}}_{2}\text{O}\left(\text{l}\right)\to {\text{MA}}_{4}{\text{PbI}}_{6}*2{\text{H}}_{2}\text{O}\left(\text{s}\right)+3{\text{PbI}}_{2}\left(\text{s}\right)$$ (1)

$${\text{MAPbI}}_{3}\left(\text{s}\right)+{\text{H}}_{2}\text{O}\to {\text{PbI}}_{2}\left(\text{s}\right)+\text{MAI}\left(\text{solution}\right)$$ (2)

$${\text{MAPbI}}_{3}\left(\text{s}\right)\to {\text{PbI}}_{2}\left(\text{s}\right)+\text{MAI}\left(\text{g}\right)$$ (3)

Perhaps similarly likely is the chemical decomposition of MAPbBr_3_ into PbBr_2_ and MABr in the presence of moisture, e.g., water vapor in air or water dissolved in solution (corresponding scenarios for MAPbI_3_ are shown in Eqs 2 and 3). It has been shown that MAPbI_3_ films decompose under annealing, leading to desorption of the MAI species at the grain boundaries and resulting in PbI_2_-rich boundaries, a mechanism also possible in MAPbBr_3_, and possibly explaining the presence of more PbBr_2_ on the *as-is* surface compared to the *cleaved* surface.[34] Contamination in solution may also be exacerbated in a sol-gel process as the surface of precursor colloids which assemble in the final solidification process can be pre-contaminated, leading to incorporation of such contaminants into the bulk of macroscopic crystals.

### 2.2. Bromine-vapor treatment

Br-deficiency and, therefore, a sub-stoichiometry in MAPbBr_3_, is obviously expected to result in Pb^2+^ cations, which can then trap electrons forming Pb°.[22, 35] Pb° has been suggested to pin the Fermi level near the conduction band minimum (CBM) resulting in an *n*-type character of perovskites.[26, 36, 37] Our Kelvin-probe measurements (S3 Fig) suggest a slightly reduced work function for the *as-is* crystal indicative of a Fermi level pinned near the CBM due to a high trap density, which agrees with the 12% higher Pb° content for the *as-is* crystal, as determined from XPS. In fact, Hall measurements (discussed later) suggest that the initial n-character of the *as-is* crystal converts into a p-character upon *cleaving*. Our findings are commensurate with a prior report on triiodide-based perovskite crystals where a work function increase upon cleaving was linked to reduced trap states in the bulk.[38] Angle-resolved XPS was recently employed to reveal a ca. 30% increase in the relative amount of Pb° on the top surface of *as-is* MAPbBr_3_ (accessed using shallow-angle emission) versus the bulk (accessed using normal emission).[26, 39] In light of this earlier report, our findings affirm that Pb° is present in the crystal bulk, albeit gets enriched at the surface of as-is crystals.

With sufficient literature suggesting that Pb° behaves as carrier traps resulting in exciton quenching, and our XPS data finding that *ca*. 10% Pb in the bulk of our crystals is Pb°, we were tempted to find a facile way to suppress Pb° and enhance charge transport in our crystals. One way of realizing this is to supply Br which can compensate for the Br deficiency, coordinating with Pb° and increasing the Br:Pb ratio. This has been demonstrated recently in the context of MAPbI_3_, when Zhang and co-workers employed hypophosphorous acid in the precursor solution to reduce the Pb°.[40] The treatment, carried out in the solution-phase during synthesis of MAPbI_3_ films, effectively increased the I:Pb ratio, suppressed metallic Pb and enhanced the PL, resulting in optoelectronically superior semiconducting films. Around the same time, Cho and co-workers reported an enhancement in current efficiency of their MAPbBr_3_ light-emitting diodes by preventing formation of Pb°.[7] The authors employed a higher molar concentration of MABr in the precursor solution resulting in significant PL improvement.[7]

Seeking process-simplicity, we were interested in developing a Pb°-suppression protocol that can be applied on as-grown bulk crystals *without* intervening in the crystal growth step. We took the view that sol-gel processes leading to the solidification of hybrid perovskite crystals through colloidal self-assembly and solvent removal may have space-filling challenges and require pathways for the trapped solvent to escape through during crystal growth. While this is likely to occur via grain boundaries in polycrystalline films,[9, 10, 41] the same cannot be said for macroscopic crystals. In the latter case, a growth front of finite thickness forms between the solid MAPbBr_3_ crystal and the precursor solution and moves during inverse temperature crystallization, forming opposing steady-state gradients of solvent and solute concentrations, as well as a gradient of density. The solvent is released by the colloidal sol-gel precursor in order for the solute to incorporate the growing MAPbI_3_ crystal. We hypothesize the escaping solvent may leave behind nanoscopic defects and porosities which subsequently allow the diffusion of gases and vapors back into the bulk. Besides, as is well-known in metal oxides and oxide perovskites, gas diffusion is possible through oxygen vacancies.

**Fig 2:**
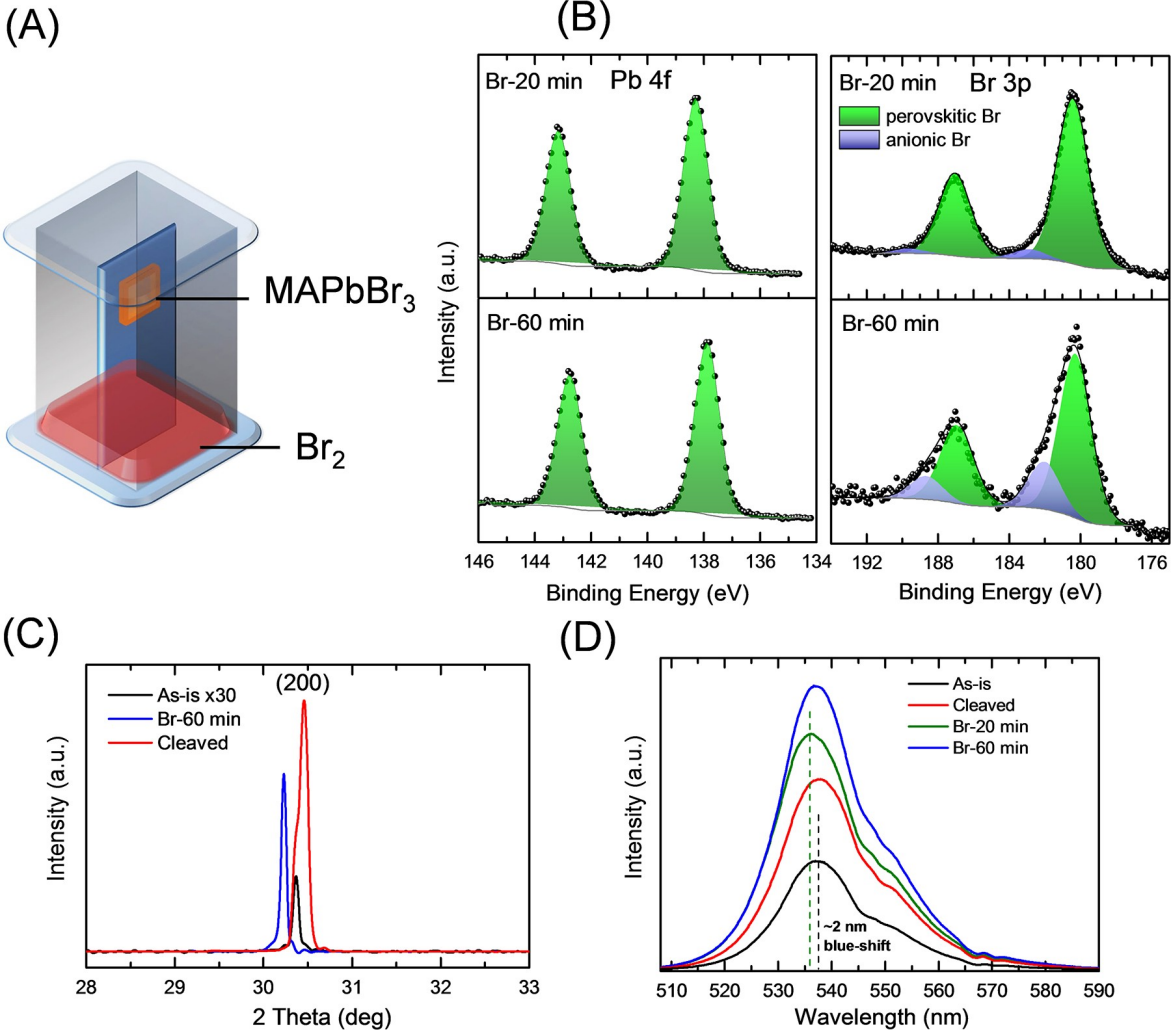
Bromine-vapor treatment. (A) Schematic showing the treatment process (B) XPS data showing the Pb 4f core peak from the top surface of the brominated crystals. Suppression of the Pb° peaks is found (C) XRD of the various MAPbBr_3_ crystals showing the (002) scattering peak shift (D) PL peaks for the various conditions. A blue-shift of *ca*. 2 nm is observed after Br-vapor treatment.

In light of these possibilities, we devised a facile and single-step Br-vapor treatment which should be capable of diffusing through defects, and vacancies (Fig 2A), and perhaps even penetrate well into the bulk material as does intercalation, for instance, into layered materials such as graphite and few layer graphene.[42, 43] As-grown and cleaved MAPbBr_3_ crystals were exposed to Br_2_-vapors for a duration ranging from a few tens-of-minutes to an hour. A 20-min treatment resulted in a Br:Pb ratio of *ca*. 3, as determined by XPS, and successfully removed Pb° species from the surface and bulk of the crystal, as verified by cleaving the same sample. Shift of the Pb 4f to lower BE by *ca*. 500 meV (Fig 2B) is consistent with the picture of *p*-doping (unpinning of the Fermi level). Fig 2B also shows the Br 3p peaks where a higher BE component (blue) develops upon 20-min bromination, becoming stronger with 60-min bromination. No changes are observed in Pb 4f peak as the treatment time is increased from 20 to 60 min. It is important to discuss here the nature and origin of the higher BE components in Br 3p, which are absent in spectra taken on *as-is* surfaces (S4 Fig). In recent work on bromination of graphene,[42, 44] these higher BE shoulders have been ascribed to anionic Br (Br^-^). The species was observed upon bromine intercalation in graphene sheets and was found to form via extraction of electrons from nearby electron-rich centers resulting in *p*-doping. In the current context, we postulate that bromination serves dual roles: (i) bromine incorporated in the perovskite host lattice accepts electrons from metallic lead, passivating Pb° and restoring stoichiometry; (ii) once stoichiometry is restored, further bromination (60-min treatment) results in an enhanced anionic Br content pushing the Br:Pb ratio to 3.3 and, probably increasing hole concentration, as shall be revealed later. We have confirmed the successive increase of anionic Br with vapor exposure time, by analyzing Br 3d peaks as well (S5 Fig).

Interestingly, similar findings have been made very recently for iodine vapor exposure of MAPbI_3_ films,[39] where the authors have observed *p*-doping resulting in enhanced electrical conductivity. The authors have argued that *p*-doping occurs as a result of suppression of iodide vacancies. In fact, the authors have used angle-resolved XPS to prove that iodine exposure results in uniform bulk doping of the perovskite rather than surface adsorption. Encouraged by these results, we carried out angle-resolved XPS on our MAPbBr_3_ crystals for the *as-is*, *cleaved* and brominated samples. XPS was measured at normal- (90 degree) and grazing- (25 degree) emission with respect to the crystal plane and the relative bromine content was determined. Following the Cahen group’s report,[39] normal emission highlights the *bulk* chemical composition, while grazing emission is highly *surface* sensitive. S2 Table summarizes the data and clearly demonstrates that the bulk/surface ratio for the relative bromine content remains unaltered by bromine vapor exposure, suggesting that there is no excess bromide on the surface. For the extended exposure for 60 mins (leading to maximum improvement in charge transport), however, the bulk/surface value is higher indicative of a significantly higher bromine incorporation in the bulk.

XRD analysis (S6 Fig) shows the scattering signatures of (001), (002) and (003) planes of cubic MAPbBr_3_ in all the samples,[12, 45, 46] suggesting that the host lattice structure is preserved. However, we observe an expansion of the lattice in the [001] direction. Further analysis of the concomitant (002) peak (Fig 2C) informed us of an apparent crystallinity enhancement as well—indicated by a dramatic increase of the diffraction intensity—in addition to a possible Br incorporation-led lattice expansion. The (002) planes appear to diffract coherently throughout the crystal with a new interplanar spacing, upon Br incorporation. Probing depth for this peak was estimated using X-ray optical constants and Bragg angle corresponding to the (002) plane and was found to be several microns (*ca*. 8 μm), directly implying that incorporation is a bulk process. The incorporation of Br into the crystal bulk was further confirmed by X-ray fluorescence (XRF) measurements, with probing depths of several hundreds of microns. Br:Pb ratio was measured by XRF: first on the top surface and then in the bulk (accessed by cleaving the crystals) of the brominated crystals. The brominated crystals retain their increased Br:Pb ratio in the bulk (S7 Fig).

Analysis of (002) peak shifts upon bromination (Fig 2C) suggests that the lattice parameter of MAPbBr_3_ increases to 5.910 Å from 5.885 Å for the *as-is* crystal. The value of 5.910 Å is close to the reported value (5.920 Å^-1^) for MAPbBr_3_.[12, 45, 47] This corresponds to a unit cell volume expansion by ~ 1.3% upon bromination. We measured the change in crystal volume using an optical microscope and found that it increased by ~ 11% upon a 60-min bromine vapor exposure. The significant discrepancy between the two values can be ascribed to factors such as deformation and strain in the crystal undergoing bromination that can exaggerate the volume expansion due to bromine-incorporation. Additionally, incorporation and adsorption of Br on the internal vacancies and porosities of a macroscopic crystal can result in volume expansion of the macroscopic crystal without incurring a lattice expansion. Taken together, these results suggest a combination of bromine-incorporation into the perovskite lattice as well as adsorption onto pore surfaces within the macroscopic crystal.

It is worth highlighting that we do not detect any presence of a crystalline Pb° phase in XRD measurements performed on the as-prepared and cleaved crystals. This is consistent with the colloidal sol-gel nature of perovskite crystallization as established previously.[48] The sol-gel ink is a complex between the precursor and the solvent. Sub-stoichiometric species can be produced through its solidification.[9, 10] Pb° is expected to form as a side-product—most likely a chemical variation of one of the chemical components (MABr/PbBr_2_)–and locally incorporate the perovskite rather than form a well-defined and crystallographically coherent phase.

Although no change in the optical band gap is found upon bromination (S8 Fig), we observe significant photoluminescence (PL) enhancement upon Br-treatment, as shown in Fig 2D, providing clear indication of significantly suppressed trap states. Given its wider bandgap compared to the usually used MAPbI_3_, MAPbBr_3_ is a strong candidate as a top cell in perovskite tandem solar cells, and can in itself lead to high open-circuit voltages (*V*_*OC*_). Improvement of PL for our brominated MAPbBr_3_ crystals is, therefore, an encouraging result in this direction. Increased radiative recombination is suggestive of suppressed trap states, which can otherwise lead to unwanted *V*_*OC*_-losses in a solar cell. For this promise to hold, the developed bromination recipe needs to be translated to device-relevant MAPbBr_3_ thin films, which is beyond the scope of the current paper. We note, however, that there has been recent interest in solar cells that employ ~20 μm thick perovskite single crystals as the active layer.[49, 50] Given the suppressed trap states and longer carrier diffusion lengths in single crystals, these cells have shown excellent carrier extraction. We believe that the Br-treatment developed in this paper is directly applicable to such single crystal solar cells for device performance improvements.

We also note a blue-shift of *ca*. 2 nm for the 60-min treated crystal compared to the untreated crystal. Our findings closely agree with studies on solution-phase Br incorporation in perovskite nanocrystals where a blue-shift and an associated enhancement was observed in PL intensity.[51] In addition, a recent report highlights the role of redox processes in perovskites (Pb^+2^ → Pb°) and demonstrates that Pb° species form deep traps in MAPbI_3_ resulting in PL quenching. These results point toward the deleterious role Pb° plays in electrical transport and that removal of this species is expected to enhance charge transport.

### 2.3. Charge transport enhancement

**Fig 3:**
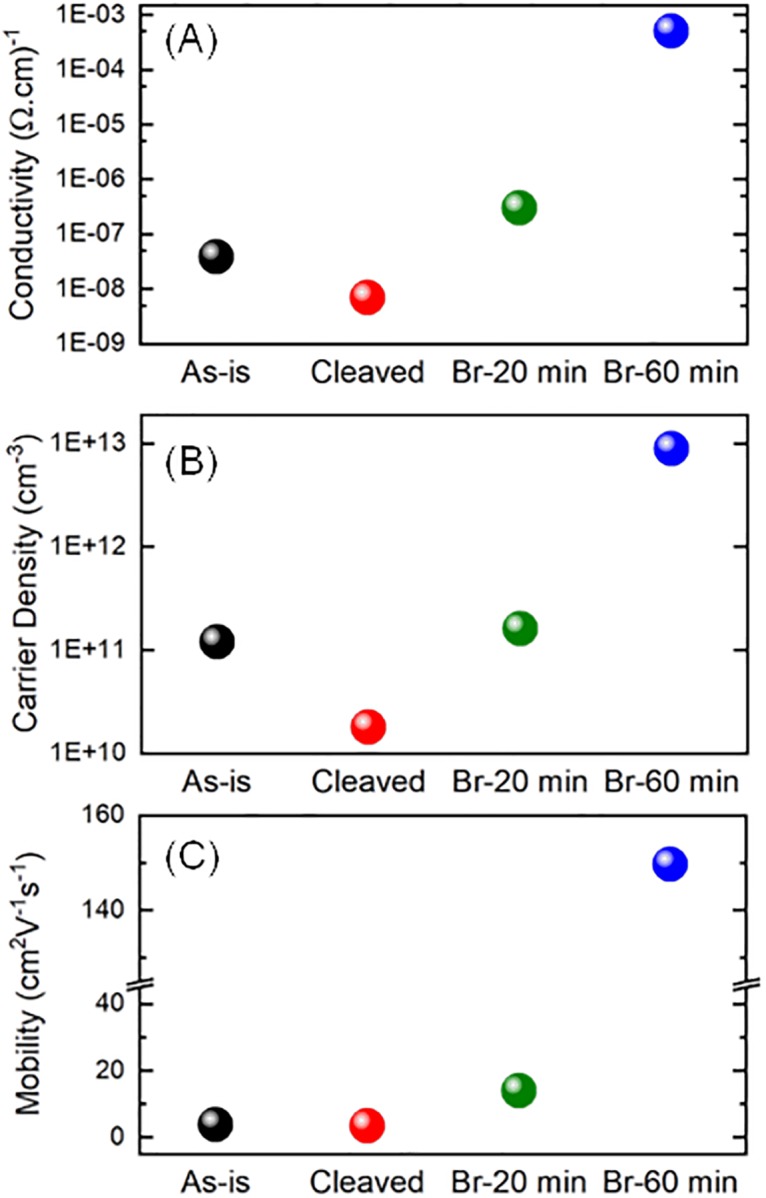
Charge transport enhancement. (A) Conductivity, (B) Carrier density, and (C) Mobility, of the untreated and Br-treated crystals obtained from Hall measurements. The measurements reveal enormous gains in charge transport. ‘Br-20 min’ and ‘Br-60 min’ represent 20-min and 60-min bromine-treated samples, respectively. Values are averages over 4–5 crystals (see S3 Table for details).

Hall-effect measurement data for untreated and Br-treated samples are shown in Fig 3 (and S3 Table). The trends reveal massively enhanced charge transport upon exposure to Br-vapor. Interestingly, charge transport is found to improve further beyond the 20 min Br-treatment by which point Pb° is already completely passivated. This confirms our above proposition that the extra anionic Br is, in fact, responsible for hole concentration enhancement.

Resistivity of the crystals decreases by four orders of magnitude indicating a 10000-fold enhancement of electrical conductivity. This entails a *ca*. 100-fold free carrier density increase to *ca*. 10^13^ cm^-3^ and a *ca*. 50-fold carrier mobility enhancement (to a maximum of *ca*. 300 cm^2^V^-1^s^-1^). Importantly, the brominated crystals exhibit *p*-character compared to the *n*-type *as-is* crystal.[26, 29, 36] Time-resolved PL experiments demonstrate that the average carrier lifetimes increase by a factor of two upon bromination, providing clear experimental evidence for the suppression of the surface defect states (S9 Fig). We ascribe the enhancements in carrier mobility and carrier concentration primarily to trap passivation and doping. However, an additional effect of the treatment may very well be an enhancement of the long-range crystalline order, as demonstrated by XRD measurements. Crystallinity improvements imply a reduction of structural defects which can potentially trap charge carriers.[12, 52] Although cleaving the *as-is* surface is also found to result in a significant enhancement of crystalline order (Fig 2C), we do not observe a markedly improved charge transport for the *cleaved* crystal (Fig 3), largely owing to the fact that metallic lead species is still detected upon cleaving. Also, the cleaving process is abrasive in nature and leads to a significantly rough crystal surface, most likely resulting in carrier scattering during Hall effect measurements and possibly compromising any gains that crystallinity enhancement can have. The carrier mobility and conductivity values that the Br_2_-vapor treatment helps us achieve are notably more than those reported for device-relevant MAPbBr_3_ thin films,[53] and crystals recently used for thermoelectric applications.[54]

It is important to understand the above trends in charge transport in light of the earlier XPS findings. These trends confirm that for smaller bromination times, Pb° is successfully removed from the crystals accompanied with an increase in transport properties (conductivity, mobility and carrier concentration) while for longer bromination times, the transport properties improve further owing to incorporation of bromide interstitials that behave as acceptor defects.

Having demonstrated the presence of Br in the crystal bulk earlier on, we obviously expect enhanced charge transport throughout the bulk of brominated crystals. To confirm, we cleaved a 60-min brominated crystal and performed Hall measurements. Conductivity (10^-4^ Ω^-1^.cm^-1^), free carrier density (10^12^ cm^-3^) and carrier mobility (280 cm^2^V^-1^s^-1^) were found to be very similar to the values measured before cleaving, in line with our expectations.

## 4. Conclusion

In summary, our carefully-performed chemical compositional analysis of MAPbBr_3_ perovskite macroscopic single crystals reveals presence of Pb° besides other chemical contaminations within the bulk of as-synthesized crystals. Although amorphous C and O get significantly reduced in the crystal bulk, Pb°, a well-known charge carrier trapper, still abounds, most likely inhibiting carrier transport. We devise a single-step, facile post-synthesis Br_2_-vapor treatment protocol which takes advantage of the inherent vacancies and porosity of MAPbBr_3_ crystals to allow vapor permeation throughout the bulk of the crystal. At shorter exposure times Br_2_ vapor treatment significantly suppresses bromine vacancies and, therefore, Pb°, while at longer timescales it introduces bromide interstitials that act as acceptor defects and *p*-dope the crystals, leading to 10000-fold and 50-fold enhancements in electrical conductivity and carrier mobility, respectively.

## Supporting information

S1 Fig MAPbBr_3_ crystals used in this study, synthesized by the inverse temperature crystallization process. a). As-is crystals and b). after bromination, and subsequent electrode deposition for Hall measurements.
(DOCX)

S2 Fig XPS measurements taking at shallow-angle emission on a cleaved crystal before (black) and after (red) 1-hour of constant X-ray exposure on the same sample spot. A small Pb° shoulder is observed at lower BE in both cases, which does not change upon X-ray exposure. Also, no noticeable change is observed in the peak shape and position.
(DOCX)

S3 Fig Contact potential difference for the *as-is* (black) and *cleaved* (red) single crystals, determined from Kelvin-probe measurements. (DOCX)

S4 Fig Br 3p core level for the as-is MAPbBr_3_ crystal. Bromine is found to be present in only one chemical environment.
(DOCX)

S5 Fig Br 3d core levels of (A) as-is and (B) brominated MAPbBr_3_ crystals. Upon bromination, the peaks are found to be composed of perovskitic (green) and ionic (blue) components. An overall shift to lower BE is due to a change in the Fermi level (p-doping), upon bromination.
(DOCX)

S6 Fig XRD patterns showing the various scattering peaks. The spectrum for the *as-is* sample (pristine) has been multiple 30x due to low intensity.
(DOCX)

S7 Fig Br:Pb atomic ratio measured by XRF suggesting that Br-enhancement in the crystal bulk is retained. (DOCX)

S8 Fig (A) Normalized absorbance spectra for the *as-is* and brominated crystals. (B) Tauc plots derived from (A) indicates that the optical band gap remains unchanged.
(DOCX)

S9 Fig Time-resolved PL lifetime measurements on the as-is (black), Br-20 min (green) and Br-60 min (pink) crystals. Average carrier lifetimes are found to increase upon bromination.
(DOCX)

S1 Table XPS comparison of chemical contamination for crystals cleaved in air versus a controlled N_2_ environment. (DOCX)

S2 Table Bulk/surface ratio of the relative Bromine content for various conditions found from angle-resolved XPS. (DOCX)

S3 Table Averages of charge transport parameters from Hall measurements, with standard deviations in brackets. Also shown in the second row of each category is the best value for that category.
(DOCX)
