# Supplementary material for: Facile and noninvasive passivation, doping and chemical tuning of macroscopic hybrid perovskite crystals
Source: PLoS One. 2020 Mar 17;15(3):e0230540. doi: 10.1371/journal.pone.0230540 (PMC7077828; doi:10.1371/journal.pone.0230540)
Supplement: S1 Fig — a). As-is crystals and b). after bromination, and subsequent electrode deposition for Hall measurements. (DOCX) [file pone.0230540.s001.docx]

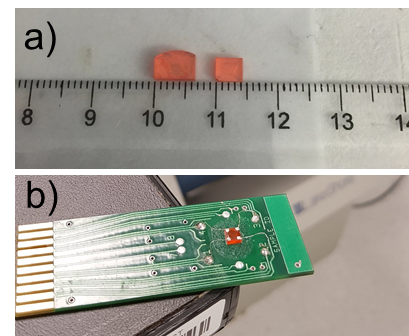


**Figure S1.** MAPbBr_3_ crystals used in this study, synthesized by the inverse temperature crystallization process. a). As-is crystals and b). after bromination, and subsequent electrode deposition for Hall measurements.
