# Supplementary material for: Facile and noninvasive passivation, doping and chemical tuning of macroscopic hybrid perovskite crystals
Source: PLoS One. 2020 Mar 17;15(3):e0230540. doi: 10.1371/journal.pone.0230540 (PMC7077828; doi:10.1371/journal.pone.0230540)
Supplement: S2 Fig — A small Pb° shoulder is observed at lower BE in both cases, which does not change upon X-ray exposure. Also, no noticeable change is observed in the peak shape and position. (DOCX) [file pone.0230540.s002.docx]

**Figure S2.** XPS measurements taking at shallow-angle emission on a cleaved crystal before (black) and after (red) 1-hour of constant X-ray exposure on the same sample spot. A small Pb^0^ shoulder is observed at lower BE in both cases, which does not change upon X-ray exposure. Also, no noticeable change is observed in the peak shape and position.
