# Supplementary material for: Facile and noninvasive passivation, doping and chemical tuning of macroscopic hybrid perovskite crystals
Source: PLoS One. 2020 Mar 17;15(3):e0230540. doi: 10.1371/journal.pone.0230540 (PMC7077828; doi:10.1371/journal.pone.0230540)
Supplement: S3 Fig — (DOCX) [file pone.0230540.s003.docx]

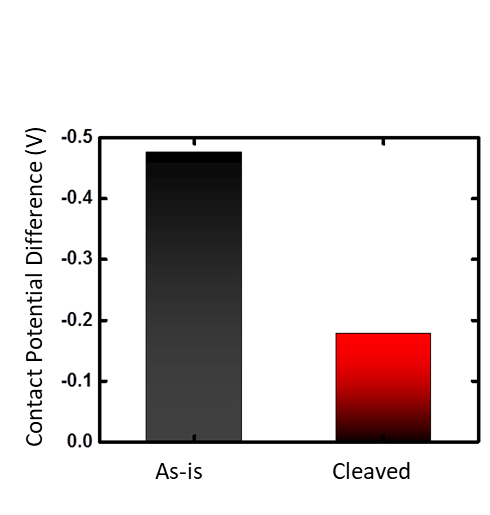


**Figure S3.** Contact potential difference for the *as-is* (black) and *cleaved* (red) single crystals, determined from Kelvin-probe measurements.
