# Supplementary material for: Facile and noninvasive passivation, doping and chemical tuning of macroscopic hybrid perovskite crystals
Source: PLoS One. 2020 Mar 17;15(3):e0230540. doi: 10.1371/journal.pone.0230540 (PMC7077828; doi:10.1371/journal.pone.0230540)
Supplement: S4 Fig — Bromine is found to be present in only one chemical environment. (DOCX) [file pone.0230540.s004.docx]

**Figure S4.** Br 3p core level for the as-is MAPbBr_3_ crystal. Bromine is found to be present in only one chemical environment.
