# Supplementary material for: Facile and noninvasive passivation, doping and chemical tuning of macroscopic hybrid perovskite crystals
Source: PLoS One. 2020 Mar 17;15(3):e0230540. doi: 10.1371/journal.pone.0230540 (PMC7077828; doi:10.1371/journal.pone.0230540)
Supplement: S5 Fig — Upon bromination, the peaks are found to be composed of perovskitic (green) and ionic (blue) components. An overall shift to lower BE is due to a change in the Fermi level (p-doping), upon bromination. (DOCX) [file pone.0230540.s005.docx]

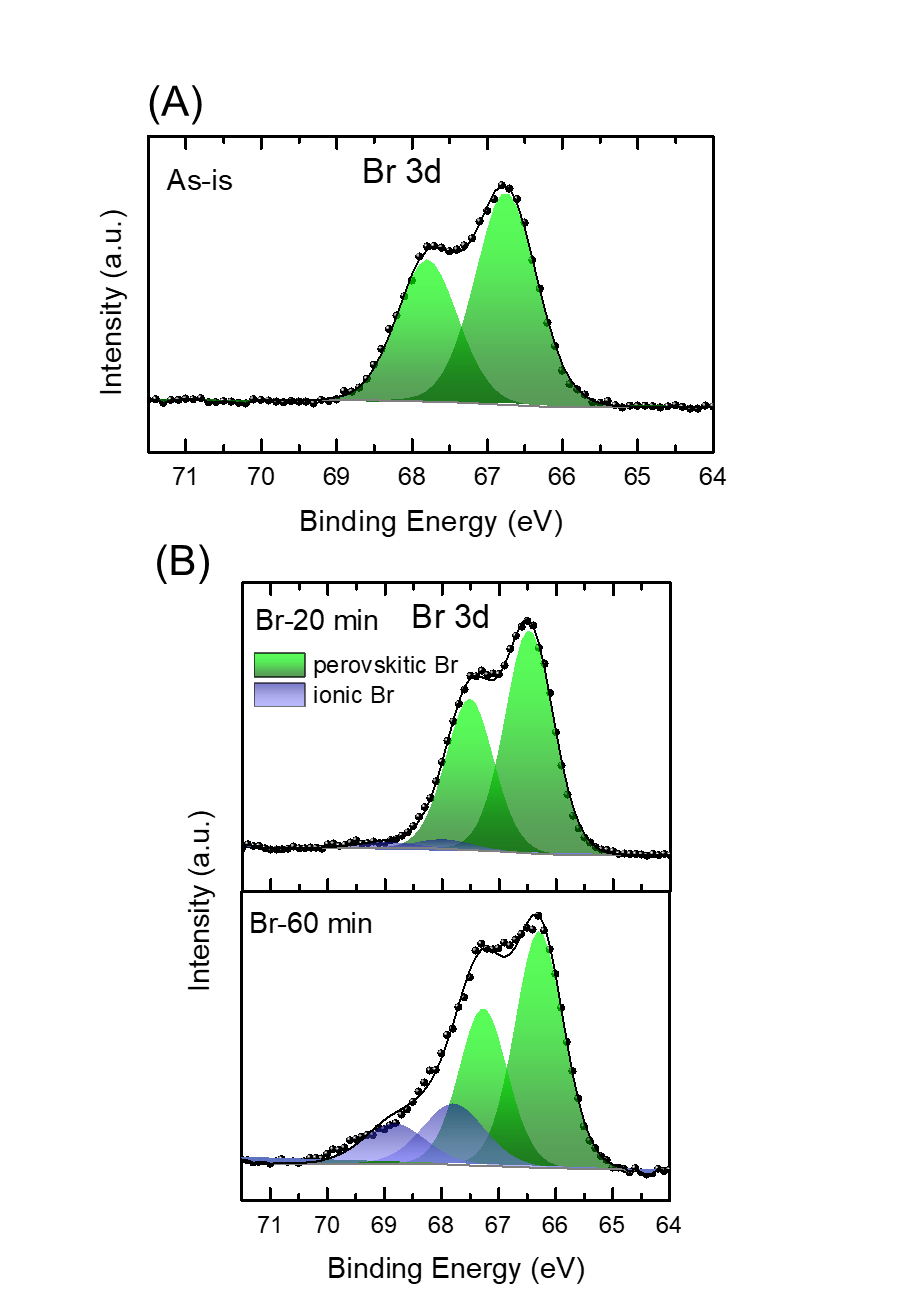


**Figure S5.** Br 3d core levels of (A) as-is and (B) brominated MAPbBr_3_ crystals. Upon bromination, the peaks are found to be composed of perovskitic (green) and ionic (blue) components. An overall shift to lower BE is due to a change in the Fermi level (p-doping), upon bromination.
