# Supplementary material for: Facile and noninvasive passivation, doping and chemical tuning of macroscopic hybrid perovskite crystals
Source: PLoS One. 2020 Mar 17;15(3):e0230540. doi: 10.1371/journal.pone.0230540 (PMC7077828; doi:10.1371/journal.pone.0230540)
Supplement: S6 Fig — The spectrum for the as-is sample (pristine) has been multiple 30x due to low intensity. (DOCX) [file pone.0230540.s006.docx]

**Figure S6.** XRD patterns showing the various scattering peaks. The spectrum for the *as-is* sample (pristine) has been multiple 30x due to low intensity.
