# Supplementary material for: Facile and noninvasive passivation, doping and chemical tuning of macroscopic hybrid perovskite crystals
Source: PLoS One. 2020 Mar 17;15(3):e0230540. doi: 10.1371/journal.pone.0230540 (PMC7077828; doi:10.1371/journal.pone.0230540)
Supplement: S7 Fig — (DOCX) [file pone.0230540.s007.docx]

**Figure S7.** Br:Pb atomic ratio measured by XRF suggesting that Br-enhancement in the crystal bulk is retained.
