# Supplementary material for: Facile and noninvasive passivation, doping and chemical tuning of macroscopic hybrid perovskite crystals
Source: PLoS One. 2020 Mar 17;15(3):e0230540. doi: 10.1371/journal.pone.0230540 (PMC7077828; doi:10.1371/journal.pone.0230540)
Supplement: S8 Fig — (A) Normalized absorbance spectra for the as-is and brominated crystals. (B) Tauc plots derived from (A) indicates that the optical band gap remains unchanged. (DOCX) [file pone.0230540.s008.docx]

**Figure S8.** (A) Normalized absorbance spectra for the *as-is* and brominated crystals. (B) Tauc plots derived from (A) indicates that the optical band gap remains unchanged.
