# Supplementary material for: Facile and noninvasive passivation, doping and chemical tuning of macroscopic hybrid perovskite crystals
Source: PLoS One. 2020 Mar 17;15(3):e0230540. doi: 10.1371/journal.pone.0230540 (PMC7077828; doi:10.1371/journal.pone.0230540)
Supplement: S9 Fig — Average carrier lifetimes are found to increase upon bromination. (DOCX) [file pone.0230540.s009.docx]

**Figure S9.** Time-resolved PL lifetime measurements on the as-is (black), Br-20 min (green) and Br-60 min (pink) crystals. Average carrier lifetimes are found to increase upon bromination.
