# Supplementary material for: Facile and noninvasive passivation, doping and chemical tuning of macroscopic hybrid perovskite crystals
Source: PLoS One. 2020 Mar 17;15(3):e0230540. doi: 10.1371/journal.pone.0230540 (PMC7077828; doi:10.1371/journal.pone.0230540)
Supplement: S1 Table — (DOCX) [file pone.0230540.s010.docx]

**Table S1.** XPS comparison of chemical contamination for crystals cleaved in air versus a controlled N_2_ environment.

| Cleaving Condition | C:Pb | O:Pb |
| --- | --- | --- |
| Air | 1.91 | 0.19 |
| GB | 2.03 | 0.26 |
