# Supplementary material for: Facile and noninvasive passivation, doping and chemical tuning of macroscopic hybrid perovskite crystals
Source: PLoS One. 2020 Mar 17;15(3):e0230540. doi: 10.1371/journal.pone.0230540 (PMC7077828; doi:10.1371/journal.pone.0230540)
Supplement: S2 Table — (DOCX) [file pone.0230540.s011.docx]

**Table S2.** Bulk/surface ratio of the relative Bromine content for various conditions found from angle-resolved XPS.

|  | Br:Pb  Normal emission (Bulk) | Br:Pb  Grazing emission (surface) | Bulk/surface |
| --- | --- | --- | --- |
| As-prepared | 1.98 | 1.72 | 1.15 |
| Cleaved | 2.33 | 2.08 | 1.12 |
| Br 15-min | 2.57 | 2.26 | 1.14 |
| Br 20-min | 2.78 | 2.45 | 1.13 |
| Br 60-min | 3.44 | 2.49 | 1.38 |
