# Supplementary material for: Facile and noninvasive passivation, doping and chemical tuning of macroscopic hybrid perovskite crystals
Source: PLoS One. 2020 Mar 17;15(3):e0230540. doi: 10.1371/journal.pone.0230540 (PMC7077828; doi:10.1371/journal.pone.0230540)
Supplement: S3 Table — Also shown in the second row of each category is the best value for that category. (DOCX) [file pone.0230540.s012.docx]

**Table S3.** Averages of charge transport parameters from Hall measurements, with standard deviations in brackets. Also shown in the second row of each category is the best value for that category.

| Sample |  |  |  |
| --- | --- | --- | --- |
|  | **Mobility**  (cm^2^V^-1^s^-2^) | **Carrier density**  (cm^-3^) | **Conductivity**  (Ω^-1^.cm^-1^) |
| As-is | 3.9 (± 1.8)  5.1162 | 1.2 (± 1.8) x 10^11^  4.2 x 10^11^ | 3.9 (± 4.6) x 10^-8^  5.8 x 10^-9^ |
| Cleaved | 3.4 (± 2.4)  5.9397 | 1.8 (± 1.1) x 10^10^  3.1 x 10^10^ | 6.9 (± 1.5) x 10^-9^  9.1 x 10^-9^ |
| Br 20-min | 14.0 (± 5.9)  19.622 | 1.6 (± 0.7) x 10^11^  2.7 x 10^11^ | 3.0 (± 0.5) x 10^-7^  3.6 x 10^-7^ |
| Br 60-min | 149.7 (± 105.7)  319.98 | 8.9 (± 11.5) x 10^12^  2.8 x 10^13^ | 5.1 (± 6.9) x 10^-4^  1.4 x 10^-3^ |
